# PTLV-1 transmission from preys to hunters in Côte d’Ivoire

**DOI:** 10.1186/1742-4690-8-S1-A89

**Published:** 2011-06-06

**Authors:** Sébastien Calvignac-Spencer, Edgard V Adjogoua, Claudia Hedemann, Chantal Akoua-Koffi, Fabian H Leendertz

**Affiliations:** 1 Research Group Emerging Zoonoses, Robert Koch-Institut, Berlin, Germany; 2 Department of Virology, Institut Pasteur Côte d’Ivoire, Abidjan, Côte d’Ivoire

## Background

Primate T-cell lymphotropic viruses type 1 (PTLV-1) infect a number of primate species, including humans. Though interspecies transmission of PTLV-1 has often been postulated, few data are actually available about possible PTLV-1 transmission among truly sympatric primate species.

## Material and methods

We investigated the questions of the prevalence and genetic diversity of PTLV-1 in wild red colobus monkeys (Piliocolobus badius badius), wild chimpanzees (Pan troglodytes verus) and humans (Homo sapiens) in the area of the Taï National Park in Côte d’Ivoire, using serological and PCR-based methods.

## Results and discussion

About 50% red colobus monkeys, 70% chimpanzees and 1% humans were PTLV-1 positive in serology and PCR. Strains detected in red colobus monkeys belonged to two main subtypes which were also found to infect sympatric chimpanzees and humans. The latter primate species could therefore have acquired those subtypes through contact to blood and organs of red colobus monkeys, which they frequently hunt and “butcher”. In addition, strains belonging to the classic, human specific Cosmopolitan subtype were detected in humans while strains belonging to the PTLV-1b subtype - already found in chimpanzees from various locations and therefore possibly widespread in the species - were detected in chimpanzees. All in all, these results point towards the possibility of co-infection of primates with both species specific (potentially adapted) PTLV-1 subtypes and other PTLV-1 subtypes acquired from sympatric primate species through hunter–prey relationships.

